# A diagnosis of conflict: theoretical barriers to integration in mental health services & their philosophical undercurrents

**DOI:** 10.1186/1747-5341-5-4

**Published:** 2010-02-04

**Authors:** Nathan M Gerard

**Affiliations:** 1 Columbia University, Teachers College, Program in Social-Organizational Psychology, 525 W 120th Street, New York, New York 10027, USA

## Abstract

This paper examines the philosophical substructure to the theoretical conflicts that permeate contemporary mental health care in the UK. Theoretical conflicts are treated here as those that arise among practitioners holding divergent theoretical orientations towards the phenomena being treated. Such conflicts, although steeped in history, have become revitalized by recent attempts at integrating mental health services that have forced diversely trained practitioners to work collaboratively together, often under one roof. Part I of this paper examines how the history of these conflicts can be understood as a tension between, on the one hand, the medical model and its use by the dominant profession of psychiatry, and on the other, those alternative models and practitioners in some way differentiated from the medical model camp. Examples will be given from recent policy and research to highlight the prevalence of this tension in contemporary practice. Part II of this paper explores the deeper commonalities that lay beneath the theoretical conflict outlined in Part I. These commonalities will be shown to be apart of a captivating framework that has continued to grip the conflict since its inception. By exposing this underlying framework--and the motivations inherent therein--the topic of integration appears in wholly different light, allowing a renewed philosophical basis for integration to emerge.

## Introduction

In the UK there has been much talk of "integrating" mental health services in recent years--a topic usually couched within a broader government agenda of assimilating health and social care. Parallel movements towards service integration can be found in North America [1] and throughout the developed and developing world [2]. In the UK, pathways towards integration have been paved by the Health Act Flexibilities (1999), which removed financial and legal constraints hindering service integration, and the Health and Social Care Act (2001), which created Care Trusts aimed to deliver a whole spectrum of services within a single organization.

The topic of service integration gains much of its appeal by appearing to make sense virtually "across the board," from the politicians and commissioners focused on partnerships and integrated budgets, to the practitioners focused on integrated working and service delivery, right down to the service user accessing a more convenient and reliable set of services provided by a "team" of professionals. Indeed, service integration has repeatedly been promoted in this sweeping manner [3-5].

Those in the midst of such integration, however, realize that it carries a number of conflicts, many of which are hardly new. One longstanding conflict poised to come to the fore by recent integration measures lies within *divergent theoretical orientations *towards the phenomena being treated. By "theoretical orientations" I wish to connote those views generally held among practitioners, either as a result of educational training or area of work (or both). Nowhere is this arguably more prevalent than in the formation of "community mental health teams" (CMHTs)--a hallmark of service integration over the past decade--whereby diversely trained practitioners are placed under one roof and, ideally, in regular contact with each other to exchange ideas and skills. As promising as this multidisciplinary, team-based approach may seem, the reality within such teams is often a cacophony of professional opinions, reinforced by deep-seated theoretical orientations [6].

This paper examines the philosophical substructure to the theoretical conflicts that permeate contemporary mental health care in the UK. Such conflicts have a tendency to overlay themselves onto philosophical chasms; by understanding these deeper differences--and their commonalities--new light may be shed on supposedly intractable barriers of thought or opinion.

## Part I

### A Diagnosis of Conflict

For many, it comes as no surprise that the field of mental health is plagued with division in both theory and practice. As psychiatrist Anthony Clare (2002) notes, "many of us in the field cannot agree on 'what we are doing', let alone 'where we are doing it' and 'why'." And it is these and other questions which, as Clare states, "still plague psychiatry two centuries on from the dawn of the scientific revolution...because psychiatry, of all the branches in medicine, is concerned with the most intricate and challenging questions concerning human life, namely the relationships between brain and mind, between genes and environment, between the individual and the group, between family and society, between the transcendent and the mundane" ([7], p.xv).

The community mental health team (CMHT)--a team comprised of psychiatrists, psychologists, social workers, community psychiatric nurses, occupational therapists, and other specialized workers--is one such setting where a diversity of views manifests in practice. Despite a diversity of views seeming beneficial, one clear concern has arisen from those practitioners trained within a "social orientation"--predominantly social workers--who fear that the "social perspective" and "social values" could lose their importance in the new multidisciplinary setting. This fear is exacerbated by integration measures taking place "higher up" with the formation of Care Trusts, which, despite aiming to deliver a spectrum of services under a single organization, commission such services almost exclusively through primary care [8].

The "social orientation" has recently been clarified by the Social Perspectives Network (SPN)--a UK organization comprised of academics, practitioners, policy makers and service users that takes as its focus the clarification of social models and their implementation in practice. SPN is quick to point out that the social orientation is not merely to be conflated with practical issues such as benefits and housing. Instead they clarify two core principles of the social orientation to mental disorder (or what they also refer to as "mental distress"):

In one sense, mental distress may be seen as a reaction to a range of social circumstances and relationships (past and present) that may be experienced as painful, contradictory, unjust, excluding or oppressive, and where no other avenue for resolution appears to present itself. In this sense, it may often link with issues of powerlessness and loss. In another sense, what may be seen as the manifestations of mental distress, such as voice hearing or self harming, may also be understood as the best available set of coping or survival strategies that a person may be able to access, given their particular history and social circumstances. In this sense, distress may paradoxically be seen as a reflection of people's resourcefulness and ingenuity ([9], p.3-4).

Thus, from the social orientation, expressions of mental disorder are seen as meaningful responses to stress in a person's life; moreover, such stress is relative to the given environment in which the person is embedded [9].

Not surprisingly, the social orientation is often both compared and promoted in opposition to a medical orientation. The archetypal medical model is portrayed as viewing mental disorder like all other medical illnesses and, hence, biological, chemical or physical in origin. As Bolton & Hill (1996) point out, there is an inherent a priori inference in this model that "psychological disorder is the breakdown of psycho-logic...of meaning, rationality, and so on, and beyond this limit we apparently have to abandon our normal intentional forms of explanation...and posit instead causal processes at the biological level which disrupt normal processes" ([10], p. 26). The medical orientation has been classically uninterested in meaning and meaningful explanations since these do not fall within the domain of the natural sciences, which have as their subject matter the objective, observable, causal and ultimately physical world [11,12].

Historically, this difference between the medical and social orientations was first theoretically articulated in psychiatry by Karl Jaspers (1913) with his celebrated distinction between meaningful and causal connections. As is well known, for Jaspers, this distinction implied a splitting of tasks for psychiatry [13]. The drawing of meaningful connections required the psychiatrist to assume the role of phenomenologist, charting the mental states of others through empathic understanding. Jaspers differentiated such a practice from the drawing of objective, causal connections between "brain-behavior" relationships from an external perspective. At the time of Jaspers' writing, this distinction was reinforced by a larger assumption pervading the social ethos that the realm of meaning was distinct from the realm of physical causes, as exemplified by the emergence of the new *Geist*, or human, sciences as distinct from the natural sciences [10].

Where the field of mental health would go with Jaspers' distinction proved formative and controversial. Although Jaspers himself held "even handedly" to the two projects of psychiatry, his contemporaries would arguably see them as mutually and radically opposed, whereby one did either one or the other, but rarely both [14]. This split was largely fueled by two implicit assumptions embedded within the discipline: namely, that the move from understanding to explaining meant a drop down " below" the mental level and into the biological and physical realms of brain and behavior; and secondly, that there was a *necessary link *between the belief in the inaccessibility of empathic understanding and a belief in biological or physiological causation [15]. Combined, these assumptions worked to imply that the move from understanding to explaining was a move that ruled out, as if by default, any more meaning and method in madness.

As Bolton (1997, 2004) makes clear, such tension reached its breaking point in the 1960s and 1970s, with medical psychiatry aligning with scientific behavioral psychology to champion the methodology of the natural sciences and psychoanalysis aligning with antipsychiatry to champion meaning, values, and the methodology of the social sciences [14,16]. In particular, antipsychiatry would call into question medical psychiatry's benignly objective and sub-personal status of mentally disordered phenomena, emphasizing the value-laden, socially constructed nature of these same phenomena. According to antipsychiatry, it was in value-laden culture where one was to find the true answers to the suffering associated with, what Thomas Szasz (1962) would call, "problems in living" [17].

### More of the Same

Some may now believe that the field has largely moved on since this period of crisis, through various concessions made on either side of the divide, along with wholly new paradigms being made available (cf. proponents of the biopsychosocial [18,19] and cognitive behavioral paradigms [10]). Nevertheless the issues that defined this historical conflict continue to resurface in various guises. This section will examine two domains where this conflict has reemerged--public policy and academic research.

The debate between the medical and social orientations has carried over to the policy context with the passage of the Disability Discrimination Act in the UK (1995) - an act that legally mandates certain rights for people diagnosed with mental disorder. On the one hand, this act seems to carry an implicit social orientation in the way it places legal requirements on employers and businesses to make "reasonable adjustments" to the work force environment through tackling those factors that make a disability "disabling" [20]. But the DDA has also been criticized by civil rights groups as implicitly "medical" in the way it defines disabled people as people with certain "limitations" that restrict their day-to-day living instead of attributing such restrictions to society [21].

Another area of UK policy where this conflict manifests is in clinical governance, in particular with the recent formation of the Social Care Institute of Excellence (SCIE) in the shadow of the National Institute of Clinical Excellence (NICE). Interestingly, both of these agencies promote the implementation of "evidence-based practice," but view the topic of what constitutes "evidence" through significantly different lenses. The SCIE promotes the use of qualitative evidence and the methods of the social sciences, while the NICE promotes quantitative evidence and the methods of the natural sciences. Furthermore, SCIE, in keeping with the social orientation, proclaims to "have a *service user focus*," "promote *empowerment *and *change*," and "be *independent *in research and findings" [22]. This contrasts with the NICE agenda to produce "*clinical *guidelines on the appropriate *treatment *of people with *specific diseases and conditions *within the NHS" [23]. The clear differences in language here is seemingly yet another tool in which to distance the medical and social orientations. The practical impact of these governing bodies is powerful, for as one socially-orientated researcher points out, the consequences of an "overly medical" approach to governance "is that resources are directed into impairment-related research and intervention, whereas scant resources are channeled into social change for the inclusion of people with social disabilities... [An] example is research within gene therapy that strives to 'cure disability' while ignoring the social and cultural factors that make not walking, hearing, seeing etc. into a problem" [20].

### The Colombo et al. study

Theoretical conflicts in contemporary mental health service settings are now becoming a topic of academic research. Precursors in this area include investigations into the differences between formal medical principles found in the literature and practitioners' assumptions [24] and the power relations between practitioners themselves and between practitioners and service users [25,26].

Of particular interest is a recent study carried out by a team of psychiatrists, sociologists and philosophers at Warwick University that proposed to examine "the influence of implicit models of mental disorder on processes of shared decision making within community-based multi-disciplinary teams," or CMHTs ([6], p. 1558). This study consisted of 100 participants responding to 12 open-ended questions concerning a case vignette. The vignette described a person whose behavior would suggest the diagnosis of schizophrenia according to the DSM-IV (see appendix for vignette). The participants were divided into five groups (20 in each group), three of which were professionals working in community mental health teams, including psychiatrists, community psychiatric nurses and social workers. The other two groups consisted of patients with long-term schizophrenia and their informal carers. The responses to the 12 questions were measured against 6 "ideal type" theoretical models identified through a literature review. These models included the medical, social, cognitive-behavioral, psycho-therapeutic, family, and conspiratorial. Each model was given a theoretical and practical component, with the former consisting of such things as "diagnosis/definition" of mental disorder, "interpretations of behavior," and "etiology," and the latter consisting of "treatment" and "prognosis."

The "ideal type" medical and social models were portrayed as follows ([6], p. 1559) in table 1:

**Table 1:** Medical and Social Models

Medical Model	
*Theoretical*	
Diagnosis/definition:	"physical health-illness continuum"
Interpretations of behavior:	"symptoms of illness as a (rough) guide to severity"
Etiology:	"physiochemical changes in the brain/genetic factors"
	
*Practical*	
Treatment:	"medical and surgical procedures, drugs etc."
Prognosis:	"many symptoms can be controlled"
	
Social Model	
*Theoretical*	
Diagnosis/definition:	"health/low stress/high stress continuum"
Interpretations of behavior:	"symptoms indicate degrees of stress"
Etiology:	"social economic stress, cultural conflict, marginal status, etc."
	
*Practical*	
Treatment:	"social change to reduce stress"
Prognosis:	"good if changes made at the social level"

When it came to interpreting the case vignette, one of the most striking findings showed that 91.3% of psychiatrists' responses matched with the "ideal type" medical model as compared with 8.8% of social workers' responses. Furthermore, when it came to within-group agreement, all 20 of the psychiatrists' responses matched with the medical model on "diagnosis/definition," "etiology" and "treatment." The social workers were more divided among themselves, perhaps reflecting the broader scope of the social orientation. For instance, when it came to those same categories among social workers (i.e. "diagnosis/definition," "etiology" and "treatment"), roughly half matched the psycho-therapeutic model, and half the social model. Interestingly, and despite this internal division, social workers were virtually unanimous in not matching the medical model on most all responses.

Another aim of the study was to investigate power-relations among practitioners through the use of semi-structured interviews based on the topic of successful and unsuccessful integrated working. In these interviews, a psychiatrist is quoted as saying "because of these ideological differences, communication is often defined in terms of a struggle between mental health and social services in which each service tries to gain control of the situation through pushing their own perspective" ([6], p. 1566). And a social worker, describing his role among the community mental health teams, states: "to be frank...to clip the wings of the psychiatrists" ([6], p. 1566). If only anecdotal, such statements give us a further glimpse into the current state of affairs among practitioners and how it tends to cleavage along medical versus social lines.

When taken together, these recent examples in both public policy and academic research suggest, in the least, that the field has not wholly transcended this theoretical tension. Instead, the various guises in which this tension manifests suggest that the field may be prisoner to its assumptions at a deeper level. A recent observation made by a group of psychologists and psychiatrists, this time in the US and associated with the Recovery Movement, succinctly captures much of the above:

Two apparently very different approaches to the treatment of mentally ill persons are emerging. The scientific, objective, evidence-based approach emphasizes external scientific reality, whereas the recovery model stresses the importance of the phenomenological, subjective experiences and autonomous rights of persons who are in recovery. The two models will conflict under many circumstances (Frese, Stanley, Kress, Vogel-Scibilia 2003, [27] p. 22).

The resurfacing of this tension may ironically be the result of integration measures themselves in so far as they have forced practitioners to work collaboratively together, often under one roof and within the same guidelines. Better understanding the roots of this conflict is crucial for assessing the future prospects of integration in theory and practice.

## PART II

### A Worthy Gesture? - "What" & "Where"

The Colombo et al. study discussed above provides a potent example of theoretical conflict in contemporary practice. Despite the clear divergences in professional opinion highlighted in this study, a subtle but important convergence can be found among all participants that is little explored; namely, all participants recognized and seemingly felt compelled to provide an account of the phenomenon in the vignette that went by the term "mental disorder." Of course, the conditions of the study would likely contribute to this shared behavior, such as the study's design, the vignette used, and the researchers and participants involved, but the behavior itself arguably resides much deeper. The historical and contemporary survey outlined above suggests that the field *does *share, if only minimally, an agreement that there is some such thing (or set of things), which seems to necessitate, if not demand, a number of responses (understanding, explaining, alleviating, containing, appreciating, etc). And yet the agreement often stops here, for to paraphrase Clare quoted earlier, we can hardly agree on "what" this something is, let alone "where" it is located [7].

In the 1970s, the debate on the concept of mental disorder was dominated by two radically different "whats" and "wheres," as indicated by the two competing terms then wrestling for status--"mental illness" versus "problem in living." The former connoted something objective and biological in constitution (a disease) which was more or less precisely situated (in an individual's mind) while the later suggested something context-sensitive and personal, or interpersonal, in constitution (a problem) and vaguely situated (in "life"). And yet, the very reason that these two terms were vigorously debated was because they purported to denote the same (or at least a very similar) phenomenon.

A number of theoretical implications can be filtered from this landmark debate, such as whether "what" we gesture at is an objective fact or social value, and whether "where" we gesture is sub-personal, personal or supra-personal; or, if that which we gesture at entails all of these things (perhaps an accurate reflection of more contemporary models), whether we can (or should) tease the theoretical points apart, setting aside the biological facts from the social values, the sub-personal from the personal from the supra-personal.

The above points are raised not to attempt an answer but to highlight what seems to be a captivating framework that encapsulates this debate along with much of the field of mental health. Central to this framework is a picture of the phenomenon of mental disorder as having a double existence, at once grounded in "exterior" reality, while at the same time also embedded in a shared "interior" life of meanings. From this picture, two major options emerge for handling this double existence: either take one side and universalize it at the expense of the other--an option exploited historically by the medical and social orientations alike (and perhaps more recently by various grand visions of cognitive science and neuroscience)--or accept this double existence as an inevitable dualism of sorts. This latter option is taken more rarely since any mention of dualism often leaves many philosophers and clinicians impatient, having claimed to "deconstruct" or "transcend" the notion long ago. Neither option, however, thinks through the motivations that give rise to this picture and that, when left unexamined, continue to regenerate the picture in its various guises.

To begin examining these motivations, a philosophical parallel can be borrowed from philosopher Charles Taylor in his discussions on the foundationalist project in philosophy [28]. This project, which finds it clearest expression in Descartes' attempt at stabilizing doubt in order to arrive at a foundation based on a clear and isolated case of reason alone, is motivated by what Taylor calls an "ontologizing of method." For Taylor (2002), this method upholds that " [t]he right way to deal with puzzles and build a reliable body of knowledge is to break the issue down into sub-questions, identify the chains of inference, dig down to an inference-free starting point, and then build by a reliable method." Taylor continues: " [o]nce this comes to seem the all-purpose nostrum for thinking, then one has an over-whelming motivation to believe that that is how the mind actually works in taking in the world" ([28], p. 109-110). This same "ontologizing of method" can be detected in the field of mental health's theoretical attempts at circumscribing the phenomenon of mental disorder in so far as this phenomenon, too, is taken as a puzzle to be dealt with by breaking down into sub-questions--in this case, a clear double existence--from which to then build back up broader theoretical orientations. The trouble with this method is two-fold: firstly--and most obvious--the process of "building back up" that entails connecting these nicely bifurcated interior and exterior words is thwarted by its very own method, which previously called for their irreconcilable split. History has shown a way around this by "building back up" one side at the expense of the other, but the end result is rarely without controversy. Secondly--and less obvious but more important--the method itself is so ingrained, as Taylor points out, that it stands as a proxy for how the mind actually takes in the world, and thus by extension, the phenomenon of mental disorder.

The abovementioned framework is highlighted not to downplay the advances made in mental health or philosophy, but rather to provide a window into the origins of the seemingly intractable conflict between the medical and social orientations. By dividing our reality into these two worlds--a world of facts independent of interpretation and a world of meanings constructed on a shared interior life--to appease our foundationalist motivations, we have not only ensured interminable conflict (byway of interminable oscillations between these two irreconcilable worlds), but have adhered to a reality far removed from our actual relationship with the phenomenon in question; that is to say, our relationship with the human being. For Taylor, " [o]ur understanding of the world is holistic from the start" ([28], p. 113). Now such a perspective would be comforting if only it were consistent with the knowledge we have of ourselves, which is often divided, fractured. In fact, as examined below, our understanding of the world and ourselves is precariously both holistic and divided; a point easily overlooked by unconsciously adhering to a framework that ignores *how *the phenomenon associated with mental disorder comes into view and *why *one feels compelled to gesture at it, and instead consumed itself with questions as to "what" and "where" it *really *is, coupled with what can or should be done about it.

### Coming Into View

There is an aspect of Karl Jaspers' work that points to this initial moment of recognizing the phenomenon of mental disorder prior to theoretical or practical elucidation. Recall Jaspers was exceptional in his ability to hold even-handedly the two tasks of psychiatry--drawing meaningful connections from within vs. drawing casual connections from without--and resisted the temptation to pin one method against the other for competitive advantage [14]. Jaspers' tolerance here arguably makes sense in the context of his later career transition into existential philosophy [29]. Intimations of an existential tone can be found in this now famous passage from Jaspers' (1913) *Allegemeine Psychopathologie*:

The most profound distinction in psychic life seems to be that between what is meaningful and *allows empathy *and what in its particular way is *ununderstandable *([13], p. 577).

This passage is famous, however, not because of any existential awe it may connote, but due to its concrete suggestion of a "barrier to understanding" in mental disorder. In light of Jaspers' distinction between meaningful and causal connections, this passage could be read as signifying the definitive end to the hermeneutic or phenomenological quest for meaning, whereby one should now move (i.e. drop down) to causal explanation.

Jaspers himself was well aware of this common move in the form of physical reductionism, which in his day was a Kraepelinian one. In particular, it was precisely Emil Kraepelin's reductionist move carried out on schizophrenia--a move which took a felt-sense of incomprehensibility and hastily translated it into an "inevitable and progressive mental deterioration," an "orchestra without a conductor"--that Jaspers hoped to avoid. Taking this historical context into consideration, Jaspers' famous quote above is just as much an attempt to resist the temptation to comprehend "schizophrenic" phenomena by physical explanation alone as it is a statement to any "barrier to understanding." In fact, Jaspers--unlike both his predecessors and successors--was seemingly able to rest in the phenomenon's utter strangeness which was "inaccessible" yet "profound." Perhaps for Jaspers, as psychologist Louis Sass (1992) suggests, "schizophrenic experience remained unintelligible not because it was beneath understanding but because it was beyond it," plausibly residing in that "ununderstandable essence of existence" that proved central to Jaspers' later existential philosophy ([15], p. 18, 409).

Put differently, Jaspers was seemingly able to recognize and appreciate the moment in which the phenomenon associated with mental disorder comes into view--the moment of recognition--prior to exercising the *need *for understanding or explaining. Parallel interest in this basic moment of human disconnect prior to theoretical elucidation can be found in philosopher Ludwig Wittgenstein's discussions on rule-following, and in particular, how one comes to recognize a disruption, or "break" in a rule. As Wittgenstein (1953) states in the *Philosophical Investigations*:

Now we get the pupil to continue a series (say + 2) beyond 1000- and he writes 1000, 1004, 1008, 1012. We say to him: "Look what you've done!"- He doesn't understand. We say: "You were meant to add *two: *look how you began the series!" ([30], para. 185)

And in the *Brown Book *(1958): "If a child does not respond to the suggestive gesture, it is separated from the others and treated as a lunatic" ([31], p. 93).

Much philosophical attention has been given to these terse and cryptic passages in an attempt to understand the nature of meaning in general, but when taken in the context of Wittgenstein's broader philosophical corpus they are revelatory with regard to our most basic interactions. Contemporary philosopher Stanley Cavell (1979), in capturing the moment of recognizing a "break" in a rule, asks the important question: "Why do we attach such importance to this that we have to make of one or other of us an outcast, or make sure that there is room in the world for both?" ([32], p. 113).

The need here, expressed by Cavell, of either having to make one of us outcasts or trying to make room for both, hints at just how the mind takes in anomalies concerning the human being in an everyday, pre-theoretical sense. From this perspective, Cavell's statement stands as microscopic portrayal of the field of mental health and its relation to the phenomenon of mental disorder: either one has to take the phenomenon as "ununderstandable" and drop to the physical stance bereft of meaning, or one tries to understand it, "make room" for it. Or one does both. But at any rate, action must be taken; one must *do something*. On the other hand, and perhaps more importantly, it stands as a portrayal of the relationship we have with ourselves. Either we have to seal this off--this anomaly--or make room for it. In both cases, action must be taken; and in both cases seemingly we are forced to either erect a boundary and seal off the (so-called) "break" from ourselves, or alternatively, resist erecting this boundary and change.

The commentary Wittgenstein and Cavell provide on our basic human interactions suggests that the "ontologizing of method" and the splitting of our theoretical orientations satisfy a more basic human need; a need often overlooked and forgotten in our more explicit gestures at the phenomenon of mental disorder, and yet--to paraphrase another famed philosopher--residing within their very warp and woof [33]. The grasping for sense that takes place in such basic moments of human disconnect and analogously in our shared basic gestures at the phenomenon of mental disorder are the first attempts at foundation. But this foundation, as Wittgenstein and Cavell point out, is built from the shifting dissonance and consonance that make up human interaction itself and thus is far from stable. In light of such instability, the formulation of a clear and captivating "picture" of the phenomenon seems understandable--natural, even--but only at the expense of the phenomenon itself. From this vantage, the medical and social orientations may themselves be an avoidance of the general phenomenon they wish to capture.

## Concluding Remarks

Recall psychiatrist Anthony Clare's diagnosis of the mental health field that opened this paper: "many of us in the field cannot agree on 'what we are doing', let alone 'where we are doing it' and 'why'." For as Clare states, "psychiatry, of all the branches in medicine, is concerned with the most intricate and challenging questions concerning human life" [7]. In light of the above investigations into the roots of these "intricate and challenging questions" a depth of perspective can now be added to Clare's loaded questions.

With regard to "what we are doing," we find that "what" we are getting at is nothing other than the human being. The idea of getting closer to something - the "where" - whether in here or out there, is only a helpful heuristic, for we need look no further than ourselves. And yet, at the same time, and in virtue of being human, we are closed off because we recognize and reinforce our separateness, from within and without. And it is this peculiar if not paradoxical human fact that both the medical and social orientations could only wish to pry open, or dissolve altogether. But it could be the very demand for a solution to this dilemma that guarantees one will never cease to make peace with it.

The field of mental health's resort to medical or social orientations, to facts or to values, as a way of gesturing at the phenomenon is, in part, a way of avoiding it. Saying this is not to suggest that we adopt a new approach or fine-tune our gestures until we finally get at "something." Rather, perhaps we should listen to and observe that very need (Clare's "why"), for it is here where we might find subtle yet revelatory insight into the human condition, prior to both theory and practice and so in life.

In light of the above investigations, the stage is now set for a renewed philosophical basis for integration to emerge that recognizes and overcomes our attachment to such deep-seated orientations and is guided by the people we serve.

## Competing interests

The author declares no competing  interests.

## About the Author

Nathan Gerard completed his Master's in Philosophy at Kings College London and is currently a Ph.D. candidate in Social-Organizational Psychology at Columbia University.

## Appendix

Colombo et al. (2003) study, case vignette [6]:

Tom Smith is a 30 year old, white male who is married with two children. During the last three days Tom has stopped eating and has said very little. A psychiatrist was called who interviewed Tom, his wife and a close family friend who has known Tom since they were at school. These interviews revealed the following facts.

About 1 year ago Tom had started to become increasingly withdrawn and preoccupied. According to both his wife and friend it seemed as though he was in a world of his own. As time went by he become less interested in his work and his children. Most of the time Tom would sit upstairs on his own, though on occasion he would become excitable and leave the house, sometimes not returning for several hours. During the last month Tom has started to express ideas which his wife finds difficult to understand.

During the interview Tom was initially reluctant to talk about his experiences but after a while he became more relaxed and said that he felt that a religious sect was putting thoughts into his mind, although he was unclear as to exactly what these thoughts were about. He has also heard members of the sect talking about him as a potential new member though he had never seen them.

According to Tom's wife, he has had no previous psychiatric problems. Furthermore, he doesn't take street drugs, drinks very little and has had no major operations since having his tonsils removed when he was 12 years old.

Tom has two brothers, one older and one younger than him, neither have had psychiatric problems nor have any other members of his immediate family except Tom's grandmother who received psychiatric treatment but no-one could remember for what reason.

Tom did not go to school until he was seven as he was described as a "delicate" child who was slow in learning to speak properly. When Tom was 8 years old his uncle, who he was very fond of, unexpectedly died. Tom was considered a very stubborn child who spent a lot of time on his own. As a teenager he lacked self-confidence and considered himself as ugly to look at.

Until recently Tom was self-employed. His small business, however, was not doing well and as a result he had a few problems paying bills and the mortgage. Tom has been married for 5 years but according to his wife they "always argued with each other."

The psychiatrist concluded the report by stating that this is all the information we have on Tom Smith.
